# Design, synthesis, and evaluation of 1, 4-benzodioxan-substituted chalcones as selective and reversible inhibitors of human monoamine oxidase B

**DOI:** 10.1080/14756366.2020.1797711

**Published:** 2020-07-23

**Authors:** Zhuo Kong, Demeng Sun, Yanmei Jiang, Yun Hu

**Affiliations:** Department of Bioengineering, Zunyi Medical University, Zhuhai, China

**Keywords:** Monoamine oxidase B selective inhibitors, reversible inhibitors, heterocyclic chalcones

## Abstract

The inhibition of monoamine oxidase B (MAO-B) could be an effective approach for the treatment of various neurological disorders. In this study, a series of 1, 4-benzodioxan-substituted chalcone derivatives were designed, synthesised and evaluated for their inhibitory activity against human MAO-B (hMAO-B). The majority of these compounds showed inhibitory activity and high selectivity. The most potent compound, (*E*)-1-(3-bromo-4-fluorophenyl)-3-(2,3-dihydrobenzo[*b*][1,4]dioxin-6-yl)prop-2-en-1-one (**22)**, exhibited an IC_50_ of 0.026 µM with a selectivity index greater than 1538. Kinetics and reversibility studies confirmed that the representative active compounds acted as competitive and reversible inhibitors of hMAO-B. The enzyme-inhibitor interactions were investigated by molecular docking studies and the rationale was provided. As these potent hMAO-B inhibitors exhibited low neurotoxicity and possessed promising drug-like properties, we believe that these active compounds could be further investigated as potential drug candidates for future *in vivo* studies.

## Introduction

Monoamine oxidases (MAO, EC 1.4.3.4) are flavin-dependent enzymesthat play important roles in the metabolism of monoamine neurotransmitters, such as dopamine, noradrenaline, serotonin. In most mammalian tissues, there are two isoforms of MAO, MAO-A and MAO-B, which share approximately 70% sequence identity. In the human body, MAO-A predominates in sympathetic nerve terminals and intestinal mucosa, whereas MAO-B is the predominant isoform expressed in the brain^1^. Although both are active towards dopamine and tyramine, the two isoforms of MAO show different substrate specificities. MAO-A preferentially catalyses the oxidative deamination of serotonin and noradrenaline, whereas the preferred substrates of MAO-B are benzylamine and β-phenylethylamine^2^^,^^3^.

**Figure 1.**
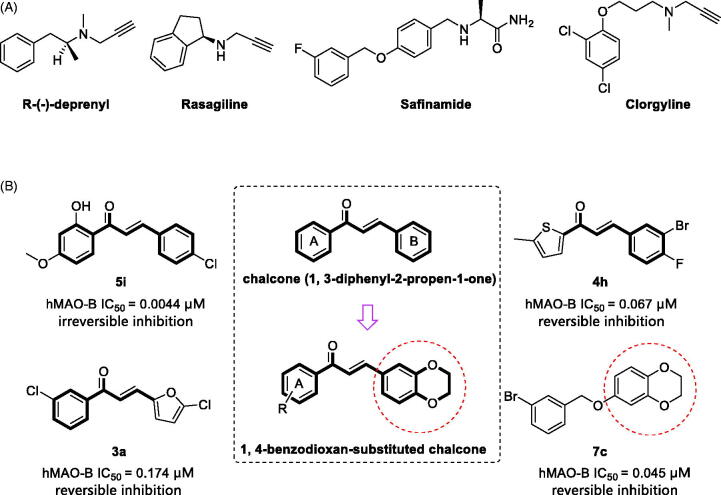
(A) Structures of irreversible and reversible MAO inhibitors in clinical use; (B) Structures, potencies and inhibition modes of previously described MAO-B inhibitors. Middle, design strategy of 1, 4-benzodioxan-substituted chalcone compounds.

Monoamines are usually degraded to aldehydes during catalytic reactions and hydrogen peroxide (H_2_O_2_) is concomitantly generated^4^. High levels of H_2_O_2_ result in the formation of reactive oxygen species (ROS), which are neurotoxic and associated with various neurodegenerative processes. The expression and activity of MAO-B significantly increase in the brain of AD and PD patients^5^. As the inhibition of MAO-B can enhance the level of dopamine in the brain and reduce the formation of ROS, MAO-B inhibitors are considered promising therapeutic agents for PD and AD^6^. Currently, two types of MAO-B inhibitors are available for the clinical treatment of PD. The first type is the irreversible MAO-B inhibitors (Figure 1(A)), such as R-(–)-deprenyl (IC_50_ = 0.0196 µM)^7^ and rasagiline (IC_50_ = 0.069 µM)^8^. The propargyl group of this class of inhibitors can form a covalent interaction with the flavin ring of MAO-B, disabling it for further catalysis^9^. These so called “suicide inhibitors” display the typical drawbacks of long-lasting enzyme inhibition, namely, *de novo* enzyme biosynthesis in the human brain and potential immunogenicity of enzyme − inhibitor adducts^10^. Reversible MAO-B inhibitors are believed to avoid these side effects and have safer profiles, as the enzyme activity can be recovered by simply terminating drug treatment. Recently, a reversible MAO-B inhibitor, safinamide (IC_50_ = 0.023 µM, Figure 1(A))^11^, has been approved for the treatment of PD. More efforts will be devoted to the development of new reversible inhibitors of MAO-B in the future.

In the search for new MAO-B inhibitors, chalcones have been used as a valid scaffold (Figure 1(B)). Chimenti et al.^12^ have synthesised plenty of chalcone derivatives based on the typical chalcone skeleton (1, 3-diphenyl-2-propen-1-one). They showed that, with the appropriate substitution, these synthetic chalcone derivatives exhibit high inhibitory potency and selectivity against human MAO-B (hMAO-B). The most active chalcone compound, **5i**, exhibits irreversible inhibition of hMAO-B with an IC_50_ value of 0.0044 µM. Interestingly, several groups^13–16^ have reported that heterocyclic chalcone derivatives, in which the phenyl rings are replaced with certain heterocyclic rings, such as furan and thiophene, are potential reversible hMAO-B inhibitors. The furanochalcone derivative **3a** and thienylchalcone derivative **4h** exhibit IC_50_ values of 0.174 µM and 0.067 µM^13^, respectively, for the inhibition of hMAO-B.

The 1, 4-benzodioxan derivatives also exhibit inhibition against MAO-B (Figure 1(B)). For example, compound **7c** inhibits the hMAO-B with an IC_50_ value of 0.045 µM^17^. It is suggested that the 1, 4-benzodioxan moiety could be useful for the design of new MAO-B inhibitors

Inspired by these results, we report the design, synthesis, and evaluation of a series of 1, 4-benzodioxan-substituted chalcone derivatives as new hMAO-B inhibitors. Additionally, the structure-activity relationship (SAR) is discussed and summarised. Hit compounds from the screen assay exhibit high inhibitory activity and selectivity, which are new reversible inhibitors of hMAO-B.

## Materials and methods

### General methods

The acetophenones and 1, 4-benzodioxan-6-carboxaldehydes were purchased from Energy Chemical Company (Shanghai, China). Other reagents and solvents were commercially available and were used without further purification. Reactions were monitored by TLC on a glass plate coated with silica gel with fluorescent indicator (GF254). Column chromatography was performed on silica gel (200 − 300 mesh). All melting points were measured on the OptiMelt automated melting point system. ^1^H NMR and ^13^C NMR spectra were recorded using TMS as an internal standard with a Bruker BioSpin Ultrashield 400 NMR system. The Purities of compounds used for biological evaluation (>95%) were determined on a Waters Ultimate HPLC system using UV monitor at 254 and 365 nm for detection. The compounds were eluted with Acetonitrile/water (0.1% TFA, w/v) in ratios of 20:80–75:25 at a flow rate of 0.3 mL/min. The Compounds purities were calculated as the percentage peak area of the analysed compound, and retention times (*t*_R_) were presented in minutes. High resolution mass spectra (HRMS) were recorded on Agilent Technologies 6530 Q-TOF. The human recombinant MAO-A and -B enzymes, Amplex Red reagent, horseradish peroxidase, R-(-)-deprenyl, rasagiline and safinamide were obtained from Sigma Aldrich.

### Synthesis of chalcone compounds

**Scheme 1.**
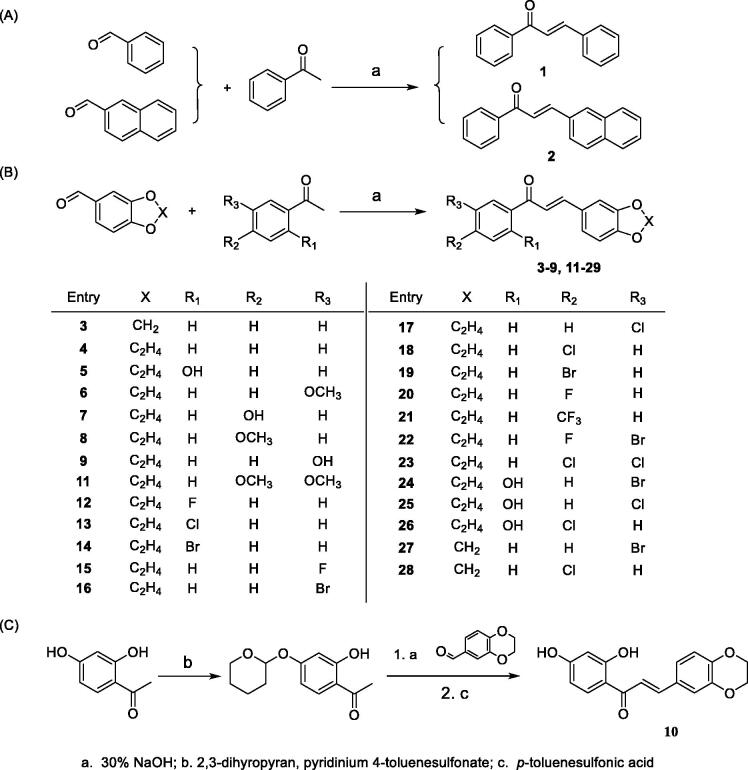
Synthesis of Compounds **1–28**.

The chalcone compounds **1**–**28** were synthesised according to the synthetic protocol outlined in Scheme 1.

#### General procedure for the synthesis of compound 1–9, 11–28

Acetophenones (1 mmol) and corresponding aromatic aldehyde (1 mmol) were dissolved in 2 mL methanol. Then 2 mL 30% NaOH was added. The mixture was kept stirred at room temperature and the reaction was monitored by TLC until completed. Under ice bath, the mixture was diluted with water and adjusted to pH = 2–3 with 5% HCl. The precipitate formed was filtered off and then purified by chromatography column with ethyl acetate/petroleum ether or recrystallization with ethanol.

#### Synthesis of compound 10

4 mmol 2, 4-dihydroxyacetophenone and 2 mmol Pyridinium 4-toluenesulfonate were dissolved in 25 mL CH_2_Cl_2_. 6 mmol 3,4-dihydro-2H-pyran was slowly added to the solution and the mixture was kept stirred at room temperature. The reaction was monitored by TLC until completed. The organic solvent was removed under vacuum and the crude product was used without further purification.

6 mmol 1, 4-benzodioxan-6-carboxaldehyde was dissolved in ethanol and mixed with the above product. Then 6 mL 30% NaOH aqueous solution was added. The mixture was kept stirred at room temperature and the reaction was monitored by TLC until completed. Under ice bath, the mixture was diluted with water and adjusted to pH = 2–3 with 5% HCl. The intermediate formed were filtered off and then purified by column chromatography. The intermediate was then dissolved in 10 mL methanol and was added with 172 mg *p*-toluenesulfonic acid. The mixture was kept stirred at room temperature and the reaction was monitored by TLC until completed. Under ice bath, the mixture was diluted with excessive water. The yellow crude product formed were then filtered off and purified by recrystallization with ethanol.

*(E)-3-(naphthalen-2-yl)-1-phenylprop-2-en-1-one* (**2**) Yield 38.8%; m.p.: 157.9–159.4 °C; UPLC purity 98.85%, *t_R_* =8.950 min. ^1^H NMR (400 MHz, DMSO-*d*_6_) δ 8.35 (s, 1H), 8.21 (d, *J* = 7.5 Hz, 2H), 8.14 (d, *J* = 8.6 Hz, 1H), 8.08 (d, *J* = 15.6 Hz, 1H), 8.01 − 7.90 (m, 2H), 7.72 − 7.57 (m, 5H). ^13^C NMR (101 MHz, DMSO-*d*_6_) δ 189.64, 144.51, 138.10, 134.42, 133.62, 133.43, 132.80, 131.17, 129.28, 129.02, 128.95, 128.19, 127.92, 127.26, 124.91, 122.85. HRMS: m/z [M + H]^+^ calcd for C_19_H_14_O: 259.1117, found: 259.1117.

*(E)-3-(benzo[d][1,3]dioxol-5-yl)-1-phenylprop-2-en-1-one* (**3**) yield 30.84%; m.p.: 117.2–118.3 °C; UPLC purity 99.18%, *t_R_* =1.697 min. ^1^H NMR (400 MHz, DMSO-*d*_6_) δ 8.18 − 8.14 (m, 1H), 7.84 (d, *J* = 15.5 Hz, 0H), 7.75 − 7.63 (m, 1H), 7.57 (t, *J* = 7.5 Hz, 1H), 7.34 (dd, *J* = 8.1, 1.7 Hz, 0H), 7.00 (d, *J* = 8.0 Hz, 0H), 6.12 (s, 1H). ^13^C NMR (101 MHz, DMSO-*d*_6_) δ 189.42, 150.06, 148.58, 144.52, 138.24, 133.41, 129.67, 129.17, 128.91, 126.42, 120.47, 108.99, 107.45, 102.13. HRMS: m/z [M + H]^+^ calcd for C_16_H_12_O_3_: 253.0800, found: 253.0883.

*(E)-3-(2,3-dihydrobenzo[b][1,4]dioxin-6-yl)-1-phenylprop-2-en-1-one* (**4**) Yield 63.52%; m.p.: 129.7–130.7 °C; UPLC purity 99.77%, *t_R_* =1.704 min. ^1^H NMR (400 MHz, DMSO-*d*_6_) δ 8.15 (d, *J* = 7.6 Hz, 2H), 7.81 (d, *J* = 15.5 Hz, 1H), 7.68 − 7.64 (m, 2H), 7.59 − 7.55 (m, 2H), 7.51 (s, 1H), 7.38 (d, *J* = 8.1 Hz, 1H), 6.94 (d, *J* = 8.3 Hz, 1H), 4.30 (q, *J* = 5.1 Hz, 4H). ^13^C NMR (101 MHz, DMSO-*d*_6_) δ 189.45, 146.35, 144.39, 144.09, 138.25, 133.40, 129.18, 128.91, 128.64, 123.52, 120.59, 117.90, 117.69, 64.89, 64.43. HRMS: m/z [M + H]^+^ calcd for C_17_H_14_O_3_: 267.1016, found: 267.1085.

*(E)-3-(2,3-dihydrobenzo[b][1,4]dioxin-6-yl)-1-(2-hydroxyphenyl)prop-2-en-1-one* (**5**) Yield 49.25%; m.p.: 120.8–122.3 °C; UPLC purity 97.49%, tR = 2.237 min, 1H NMR (400 MHz, DMSO-*d*_6_) δ 12.73 (s, 1H), 8.30 (d, J = 7.2 Hz, 1H), 7.92 (d, J = 15.4 Hz, 1H), 7.77 (d, J = 15.4 Hz, 1H), 7.59–7.55 (m, 2H), 7.41 (dd, J = 8.4, 1.7 Hz, 1H), 7.02- 6.98 (m, 2H), 6.95 (d, J = 8.4 Hz, 1H), 4.31 (dd, J = 9.8, 5.0 Hz, 4H). ^13^C NMR (101 MHz, DMSO-*d*_6_) δ 194.07, 162.57, 146.76, 145.50, 144.14, 136.73, 131.33, 128.42, 124.05, 120.99, 119.92, 119.55, 118.19, 118.01, 117.98, 64.94, 64.42. HRMS: m/z [M-H]^+^ calcd for C_17_H_14_O_4_: 281.0819, found: 281.0827.

*(E)-3-(2,3-dihydrobenzo[b][1,4]dioxin-6-yl)-1–(3-methoxyphenyl)prop-2-en-1-one* (**6**) Yield 75.1%; m.p.: 94.4–95.6 °C; UPLC purity 97.46%, *t_R_* =7.624 min. ^1^H NMR (400 MHz, DMSO-*d*_6_) δ 7.84 − 7.71 (m, 2H), 7.65 (d, *J* = 15.5 Hz, 1H), 7.61 (s, 1H), 7.56 − 7.42 (m, 2H), 7.37 (d, *J* = 8.4 Hz, 1H), 7.22 (d, *J* = 8.2 Hz, 1H), 6.93 (d, *J* = 8.4 Hz, 1H), 4.29 (q, *J* = 5.2 Hz, 4H), 3.85 (s, 3H). ^13^C NMR (101 MHz, DMSO-*d*_6_) δ 189.20, 159.99, 146.35, 144.47, 144.07, 139.71, 130.31, 128.62, 123.59, 121.43, 120.62, 119.45, 117.88, 117.73, 113.43, 64.88, 64.42, 55.82. HRMS: m/z [M + H]^+^ calcd for C_18_H_16_O_4_: 297.1121, found: 297.1134.

*(E)-3–(2,3-dihydrobenzo[b][1,4]dioxin-6-yl)-1-(4-hydroxyphenyl)prop-2-en-1-one* (**7**) Yield 29%; m.p.: 206.3–207.7 °C; UPLC purity 99.79%, *t_R_* =2.327 min. ^1^H NMR (400 MHz, DMSO-*d*_6_) δ 10.41 (s, 1H), 8.08 (d, *J* = 8.7 Hz, 2H), 7.77 (d, *J* = 15.5 Hz, 1H), 7.59 (d, *J* = 15.5 Hz, 1H), 7.47 (d, *J* = 1.7 Hz, 1H), 7.35 (dd, *J* = 8.4, 1.8 Hz, 1H), 6.93 (d, *J* = 8.6 Hz, 2H), 6.89(s, 1H), 4.30 (q, *J* = 4.7 Hz, 4H). ^13^C-NMR (101 MHz, DMSO-*d*_6_) δ 187.49, 162.48, 146.03, 144.05, 143.05, 131.54, 129.77, 128.86, 123.21, 120.65, 117.86, 117.47, 115.77, 64.85, 64.43. HRMS: m/z [M-H]^+^ calcd for C_17_H_14_O_4_: 281.0819, found: 281.0831.

*(E)-3–(2,3-dihydrobenzo[b][1,4]dioxin-6-yl)-1-(4-methoxyphenyl)prop-2-en-1-one* (**8**) Yield 43.9%; m.p.: 93.8–94.9 °C; UPLC *purity* 98.82%, *t_R_* =7.483 min. ^1^H NMR (400 MHz, DMSO-*d*_6_) δ 8.17 (d, *J* = 8.8 Hz, 2H), 7.80 (d, *J* = 15.5 Hz, 1H), 7.62 (d, *J* = 15.5 Hz, 1H), 7.49 (d, *J* = 2.0 Hz, 1H), 7.36 (dd, *J* = 8.4, 2.0 Hz, 1H), 7.08 (d, *J* = 8.7 Hz, 2H), 6.93 (d, *J* = 8.4 Hz, 1H), 4.30 (q, *J* = 5.0 Hz, 4H), 3.87 (s, 3H). ^13^C NMR (101 MHz, DMSO-*d*_6_) δ 187.68, 163.56, 146.14, 144.07, 143.49, 131.29, 131.11, 128.80, 123.37, 120.56, 117.86, 117.56, 114.41, 64.86, 64.43, 56.01. HRMS: m/z [M + H]^+^ calcd for C_18_H_16_O_4_: 297.1121, found: 297.1041.

*(E)-3–(2,3-dihydrobenzo[b][1,4]dioxin-6-yl)-1-(3-hydroxyphenyl)prop-2-en-1-one* (**9**) Yield 63.76%; m.p.: 196.8–198.5 °C; UPLC purity 99.26%, *t_R_* =6.407 min. ^1^H NMR (400 MHz, DMSO-*d*_6_) δ 9.77 (s, 1H), 7.71 (d, *J* = 15.6 Hz, 1H), 7.67 − 7.56 (m, 2H), 7.48 − 7.46 (m, 2H), 7.38 − 7.34 (m, 2H), 7.05 (dd, *J* = 8.1, 2.5 Hz, 1H), 6.93 (d, *J* = 8.4 Hz, 1H), 4.30 (q, *J* = 4.8 Hz, 4H). ^13^C NMR (101 MHz, DMSO-*d*_6_) δ 189.39, 158.14, 146.29, 144.16, 144.08, 139.70, 130.22, 128.65, 123.40, 120.78, 120.55, 119.97, 117.90, 117.66, 115.05, 64.88, 64.42. HRMS: m/z [M + H]^+^ calcd for C_17_H_14_O_4_: 283.0965, found: 283.0982.

*(E)-3-(2,3-dihydrobenzo[b][1,4]dioxin-6-yl)-1–(2,4-dihydroxyphenyl)prop-2-en-1-one* (**10**) Yield 16.45%; m.p.: 169.5–170.8 °C; UPLC purity 93.36%, *t_R_* =1.582 min. ^1^H NMR (400 MHz, DMSO-*d*_6_) δ 13.54 (s, 1H), 10.76 (s, br, 1H), 8.21 (d, *J* = 9.0 Hz, 1H), 7.83 (d, *J* = 15.4 Hz, 1H), 7.71 (d, *J* = 15.3 Hz, 1H), 7.53 (d, *J* = 1.5 Hz, 1H), 7.39 − 7.37 (m, 1H), 6.94 (d, *J* = 8.4 Hz, 1H), 6.43 − 6.40 (m, 1H), 6.30 (d, *J* = 2.0 Hz, 1H), 4.31 − 4.29 (m, 4H).^13^C-NMR (101 MHz, DMSO-*d*_6_) δ 191.94, 166.32, 165.61, 146.44, 144.14, 144.11, 133.55, 128.60, 123.77, 119.65, 117.91, 117.79, 113.47, 108.63, 103.04, 64.90, 64.42. HRMS: m/z [M + H]^+^ calcd for C_17_H_14_O_5_: 299.0914, found: 299.0919.

*(E)-3–(2,3-dihydrobenzo[b][1,4]dioxin-6-yl)-1–(3,4-dimethoxyphenyl)prop-2-en-1-one* (**11**) Yield 55.16%; m.p.: 110.0–111.1 °C; UPLC purity 94.77%, *t_R_* =6.966 min. ^1^H NMR (400 MHz, DMSO-*d*_6_) δ 7.91 (dd, *J* = 8.4, 2.0 Hz, 1H), 7.81 (d, *J* = 15.5 Hz, 1H), 7.62 (d, *J* = 13.1 Hz, 2H), 7.50 (s, 1H), 7.37 (dd, *J* = 8.4, 2.0 Hz, 1H), 7.09 (d, *J* = 8.4 Hz, 1H), 6.93 (d, *J* = 8.3 Hz, 1H), 4.30 (q, *J* = 5.1 Hz, 4H), 3.87 (d, *J* = 4.0 Hz, 6H). ^13^C NMR (101 MHz, DMSO-*d*_6_) δ 187.67, 153.56, 149.23, 146.12, 144.06, 143.41, 131.17, 128.81, 123.69, 123.39, 120.51, 117.84, 117.57, 111.34, 111.27, 64.87, 64.43, 56.23, 56.08. HRMS: m/z [M + H]^+^ calcd for C_19_H_18_O_5_: 327.1227, found: 327.1251.

*(E)-3–(2,3-dihydrobenzo[b][1,4]dioxin-6-yl)-1-(2-fluorophenyl)prop-2-en-1-one* (**12**) Yield 14.1%; m.p.: 93.7–95.4 °C; UPLC purity 98.88%, *t_R_* =7.654 min. ^1^H NMR (400 MHz, DMSO-*d*_6_) δ 7.76 (t, *J* = 7.4 Hz, 1H), 7.65 (q, *J* = 6.8, 6.4 Hz, 1H), 7.54 (d, *J* = 15.7 Hz, 1H), 7.42 − 7.23 (m, 5H), 6.92 (d, *J* = 8.4 Hz, 1H), 4.29 (q, *J* = 4.9 Hz, 4H). ^13^C NMR (101 MHz, DMSO-*d*_6_) δ 189.23, 161.81, 159.32, 146.58, 145.08, 144.10, 134.47, 134.38, 130.88, 130.86, 128.16, 127.68, 127.55, 125.25, 125.23, 124.24, 124.21, 123.27, 118.04, 117.75, 117.14, 116.92, 64.89, 64.40. HRMS: m/z [M + H]^+^ calcd for C_17_H_13_FO_3_:285.0921, found: 285.0809.

*(E)-1-(2-chlorophenyl)-3-(2,3-dihydrobenzo[b][1,4]dioxin-6-yl)prop-2-en-1-one* (**13**) Yield 36%; m.p.: 67.5–69.4 °C; UPLC purity 99.39%, *t*_R_ =7.686 min. ^1^H NMR (400 MHz, DMSO-d6) δ 7.59 − 7.51 (m, 3H), 7.50 − 7.47 (m, 1H), 7.35 − 7.29 (m, 2H), 7.24 (dd, *J* = 8.4, 2.0 Hz, 1H), 7.12 (d, *J* = 16.0 Hz, 1H), 6.90 (d, *J* = 8.4 Hz, 1H), 4.33 − 4.24 (m, 4H). ^13^C NMR (101 MHz, DMSO-*d*_6_) δ 193.44, 146.69, 146.58, 144.12, 139.35, 132.12, 130.51, 130.34, 129.67, 127.98, 127.81, 124.92, 123.45, 118.03, 117.77, 64.90, 64.38. HRMS: m/z [M + H]^+^ calcd for C_17_H_13_ClO_3_: 301.0626, found: 301.0645.

*(E)-1-(2-bromophenyl)-3-(2,3-dihydrobenzo[b][1,4]dioxin-6-yl)prop-2-en-1-one* (**14**) Yield 39.3%; m.p.: 70.3–72.2 °C; UPLC purity 99.68%, *t_R_* =7.864 min. ^1^H NMR (400 MHz, DMSO-*d*_6_) δ 7.73 (d, *J* = 7.8 Hz, 1H), 7.54 − 7.49 (m, 2H), 7.47 − 7.43 (m, 1H), 7.34 − 7.23 (m, 3H), 7.09 (d, *J* = 16.0 Hz, 1H), 6.90 (d, *J* = 8.4 Hz, 1H), 4.34 − 4.23 (m, 4H). ^13^C NMR (101 MHz, DMSO-*d*_6_) δ 194.36, 146.78, 146.70, 144.13, 141.41, 133.59, 132.12, 129.54, 128.26, 127.98, 124.75, 123.46, 119.05, 118.03, 117.76, 64.90, 64.38. HRMS: m/z [M + H]^+^ calcd for C_17_H_13_BrO_3_: 345.0121, found: 345.0138.

*(E)-3-(2,3-dihydrobenzo[b][1,4]dioxin-6-yl)-1-(3-fluorophenyl)prop-2-en-1-one* (**15**) Yield 70.4%; m.p.: 128.3–12.4 °C; UPLC purity 93.14%, *t_R_* =7.813 min. ^1^H NMR (400 MHz, DMSO-*d*_6_) δ 8.00 (d, *J* = 7.7 Hz, 1H), 7.96 (d, *J* = 9.8 Hz, 1H), 7.81 (d, *J* = 15.5 Hz, 1H), 7.68 (d, *J* = 15.5 Hz, 1H), 7.61 (td, *J* = 8.0, 5.8 Hz, 1H), 7.54 − 7.48 (m, 2H), 7.39 (dd, *J* = 8.5, 2.1 Hz, 1H), 6.93 (d, *J* = 8.4 Hz, 1H), 4.30 (q, *J* = 5.4 Hz, 4H). ^13^C NMR (101 MHz, DMSO-*d*_6_) δ 188.18, 164.02, 161.59, 146.52, 145.13, 144.09, 140.52, 140.46, 131.36, 131.29, 128.52, 125.06, 125.03, 123.82, 120.38, 120.17, 117.89, 117.79, 115.55, 115.33, 64.90, 64.42. HRMS: m/z [M + H]^+^ calcd for C_17_H_13_FO_3_: 285.0921, found: 285.0929.

*(E)-1-(3-bromophenyl)-3-(2,3-dihydrobenzo[b][1,4]dioxin-6-yl)prop-2-en-1-one* (**16**) Yield 25.53%; m.p.: 119.3–120.4 °C; UPLC purity 99.08%, *t_R_* =2.557 min. ^1^H NMR (400 MHz, DMSO-*d*_6_) δ 8.32 (t, *J* = 1.8 Hz, 1H), 8.15 (d, *J* = 7.8 Hz, 1H), 7.90 − 7.77 (m, 2H), 7.68 (d, *J* = 15.5 Hz, 1H), 7.61 − 7.47 (m, 2H), 7.40 (dd, *J* = 8.4, 2.1 Hz, 1H), 6.94 (d, *J* = 8.3 Hz, 1H), 4.30 (q, *J* = 5.3 Hz, 4H). ^13^C NMR (101 MHz, DMSO-*d*_6_) δ 188.06, 146.56, 145.26, 144.10, 140.27, 136.04, 131.41, 128.53, 127.90, 123.90, 122.76, 120.10, 117.89, 117.85, 64.91, 64.42. HRMS: m/z [M + H]^+^ calcd for C_17_H_13_BrO_3_: 345.0121, found: 345.0124.

*(E)-1-(3-chlorophenyl)-3-(2,3-dihydrobenzo[b][1,4]dioxin-6-yl)prop-2-en-1-one* (**17**) Yield 49.25%; m.p.: 125.5–127.0 °C; UPLC purity 95.22%, *t_R_* =2.366 min. ^1^H NMR (400 MHz, DMSO-*d*_6_) δ 8.20 (t, *J* = 1.9 Hz, 1H), 8.11 (d, *J* = 7.9 Hz, 1H), 7.83 (d, *J* = 15.5 Hz, 1H), 7.76 − 7.64 (m, 2H), 7.63 − 7.54 (m, 2H), 7.40 (dd, *J* = 8.4, 2.1 Hz, 1H), 6.94 (d, *J* = 8.4 Hz, 1H), 4.30 (q, *J* = 5.3 Hz, 4H). ^13^C NMR (101 MHz, DMSO-*d*_6_) δ 188.12, 146.55, 145.25, 144.10, 140.07, 134.26, 133.13, 131.15, 128.57, 128.53, 127.53, 123.89, 120.11, 117.89, 117.84, 64.91, 64.42. HRMS: m/z [M + H]^+^ calcd for C_17_H_13_ClO_3_: 301.0626, found: 301.0638.

*(E)-1–(4-chlorophenyl)-3–(2,3-dihydrobenzo[b][1,4]dioxin-6-yl)prop-2-en-1-one* (**18**) Yield 58.43%; m.p.: 102.2–103.3 °C; UPLC purity 99.59%, *t_R_* =2.383 min. ^1^H NMR (400 MHz, DMSO-*d*_6_) δ 8.18 (d, *J* = 8.5 Hz, 2H), 7.80 (d, *J* = 15.5 Hz, 1H), 7.70 − 7.60 (m, 3H), 7.38 (dd, *J* = 8.4, 2.0 Hz, 1H), 6.93 (d, *J* = 8.3 Hz, 1H), 4.30 (d, *J* = 4.1 Hz, 4H). ^13^C NMR (101 MHz, DMSO-*d*_6_) δ 188.31, 146.47, 144.89, 144.09, 138.37, 136.89, 130.84, 129.28, 128.55, 123.69, 120.23, 117.91, 117.76, 64.90, 64.43. HRMS: m/z [M + H]^+^ calcd for C_17_H_13_ClO_3_: 301.0626, found: 301.0632.

*(E)-1–(4-bromophenyl)-3–(2,3-dihydrobenzo[b][1,4]dioxin-6-yl)prop-2-en-1-one* (**19**) Yield 50.48%; m.p.: 136.7–138.2 °C; UPLC purity 99.71%, *t_R_* =2.525 min. ^1^H NMR (400 MHz, DMSO-*d*_6_) δ 8.09 (d, *J* = 8.6 Hz, 2H), 7.84 − 7.73 (m, 3H), 7.67 (d, *J* = 15.5 Hz, 1H), 7.51 (s, 1H), 7.38 (dd, *J* = 8.4, 2.0 Hz, 1H), 6.93 (d, *J* = 8.3 Hz, 1H), 4.30 (q, *J* = 4.8 Hz, 4H). ^13^C NMR (101 MHz, DMSO-*d*_6_) δ 188.52, 146.48, 144.93, 144.09, 137.21, 132.24, 130.96, 128.55, 127.56, 123.70, 120.20, 117.91, 117.77, 64.90, 64.43. HRMS: m/z [M + H]^+^ calcd for C_17_H_13_BrO_3_: 345.0121, found: 345.0132.

*(E)-3-(2,3-dihydrobenzo[b][1,4]dioxin-6-yl)-1-(4-fluorophenyl)prop-2-en-1-one* (**20**) Yield 36.63%; m.p.: 135.5–136.5 °C; UPLC purity 96.31%, *t_R_* =7.718 min. ^1^H NMR (400 MHz, DMSO-*d*_6_) δ 8.25 (dd, *J* = 8.5, 5.5 Hz, 2H), 7.81 (d, *J* = 15.5 Hz, 1H), 7.66 (d, *J* = 15.5 Hz, 1H), 7.51 (s, 1H), 7.44 − 7.30 (m, 3H), 6.93 (d, *J* = 8.4 Hz, 1H), 4.30 (q, *J* = 5.1 Hz, 4H). ^13^C NMR (101 MHz, DMSO-*d*_6_) δ 187.94, 166.67, 164.17, 146.39, 144.55, 144.08, 134.91, 134.89, 131.93, 131.83, 128.59, 123.61, 120.31, 117.88, 117.69, 116.26, 116.05, 64.88, 64.42. HRMS: m/z [M + H]^+^ calcd for C_17_H_13_FO_3_: 285.0921, found: 285.0940.

*(E)-3-(2,3-dihydrobenzo[b][1,4]dioxin-6-yl)-1-(4-(trifluoromethyl)phenyl)prop-2-en-1-one* (**21**) Yield 41.9%; m.p.: 139.0–140.2 °C; UPLC purity 96.39%, *t_R_* =8.629 min. ^1^H NMR (400 MHz, DMSO-*d*_6_) δ 8.32 (d, *J* = 8.1 Hz, 2H), 7.92 (d, *J* = 8.2 Hz, 2H), 7.82 (d, *J* = 15.6 Hz, 1H), 7.71 (d, *J* = 15.5 Hz, 1H), 7.53 (d, *J* = 2.0 Hz, 1H), 7.40 (dd, *J* = 8.4, 2.1 Hz, 1H), 6.95 (d, *J* = 8.4 Hz, 1H), 4.31 (q, *J* = 4.9 Hz, 4H). ^13^C NMR (101 MHz, DMSO-*d*_6_) δ 188.85, 146.64, 145.57, 144.11, 141.50, 132.88, 132.56, 129.67, 128.44, 126.13, 126.09, 125.67, 123.81, 122.97, 120.34, 117.94, 117.86, 64.92, 64.42. HRMS: m/z [M + H]^+^ calcd for C_18_H_13_F_3_O_3_: 335.0890, found: 335.0912.

*(E)-1–(3-bromo-4-fluorophenyl)-3–(2,3-dihydrobenzo[b][1,4]dioxin-6-yl)prop-2-en-1-one* (**22**) Yield 41.2%; m.p.: 160.1–161.6 °C; UPLC purity 97.93%, *t_R_* =8.688 min. ^1^H NMR (400 MHz, DMSO-*d*_6_) δ 8.51 (dd, *J* = 6.9, 2.1 Hz, 1H), 8.24 − 8.18 (m, 1H), 7.83 (d, *J* = 15.4 Hz, 1H), 7.67 (d, *J* = 15.4 Hz, 1H), 7.54 (d, *J* = 2.4 Hz, 2H), 7.38 (dd, *J* = 8.5, 2.0 Hz, 1H), 6.92 (d, *J* = 8.4 Hz, 1H), 4.30 (q, *J* = 4.8 Hz, 4H). ^13^C NMR (101 MHz, DMSO-*d*_6_) δ 186.79, 162.72, 160.21, 146.55, 145.28, 144.08, 136.18, 136.15, 134.39, 130.91, 130.83, 128.51, 123.94, 119.83, 117.85, 117.82, 117.67, 117.45, 109.36, 109.15, 64.91, 64.42. HRMS: m/z [M + H]^+^ calcd for C_17_H_12_BrFO_3_: 363.0027, found: 363.0040.

*(E)-1-(3,4-dichlorophenyl)-3–(2,3-dihydrobenzo[b][1,4]dioxin-6-yl)prop-2-en-1-one* (**23**) Yield 62.7%; m.p.: 156.6–157.9 °C; UPLC purity 93.35%, *t_R_* =9.358 min. ^1^H NMR (400 MHz, DMSO-*d*_6_) δ 8.40 (s, 1H), 8.11 (d, *J* = 8.4 Hz, 1H), 7.85 − 7.81 (m, 2H), 7.69 (d, *J* = 15.4 Hz, 1H), 7.55 (s, 1H), 7.40 (d, *J* = 8.4 Hz, 1H), 6.93 (d, *J* = 8.3 Hz, 1H), 4.31 (q, *J* = 5.2 Hz, 4H). ^13^C NMR (101 MHz, DMSO-*d*_6_) δ 187.18, 146.64, 145.57, 144.09, 138.35, 136.26, 132.36, 131.50, 130.79, 128.90, 128.48, 124.00, 119.80, 117.87, 64.92, 64.42. HRMS: m/z [M + H]^+^ calcd for C_17_H_12_Cl_2_O_3_: 335.0236, found: 335.0252.

*(E)-1-(5-bromo-2-hydroxyphenyl)-3-(2,3-dihydrobenzo[b][1,4]dioxin-6-yl)prop-2-en-1-one* (**24**) Yield 58.33%; m.p.: 141.8–143.7 °C; UPLC purity 98.33%, *t_R_* =9.201 min. ^1^H NMR (400 MHz, DMSO-*d*_6_) δ 12.59 (s, 1H), 8.41 (s, 1H), 7.87 (d, *J* = 15.3 Hz, 1H), 7.75 (d, *J* = 15.3 Hz, 1H), 7.67 (d, *J* = 8.4 Hz, 1H), 7.57 (s, 1H), 7.41 (d, *J* = 8.4 Hz, 1H), 6.95 (t, *J* = 9.7 Hz, 2H), 4.30 (q, *J* = 4.8 Hz, 4H). ^13^C NMR (101 MHz, DMSO-*d*_6_) δ 192.88, 161.14, 146.88, 146.15, 144.13, 138.72, 132.94, 128.35, 124.31, 123.13, 120.53, 120.12, 118.09, 117.92, 110.76, 64.95, 64.40.

*(E)-1-(5-chloro-2-hydroxyphenyl)-3-(2,3-dihydrobenzo[b][1,4]dioxin-6-yl)prop-2-en-1-one* (**25**) Yield 60.12%; m.p.: 136.2–137.3 °C; UPLC purity 98.79%, *t_R_* =3.216 min. ^1^H NMR (400 MHz, DMSO-*d*_6_) δ 12.59 (s, 1H), 8.32 (s, 1H), 7.97 − 7.71 (m, 2H), 7.63 − 7.36 (m, 3H), 6.98 (dd, *J* = 31.8, 8.5 Hz, 2H), 4.31 (s, 4H). ^13^C NMR (101 MHz, DMSO-*d*_6_) δ 192.95, 160.80, 146.89, 146.16, 144.13, 135.96, 130.12, 128.35, 124.33, 123.33, 122.48, 120.12, 120.06, 118.08, 117.92, 64.95, 64.41. HRMS: m/z [M + H]^+^ calcd for C_17_H_13_ClO_4_: 317.0575, found: 317.0593.

*(E)-1-(4-chloro-2-hydroxyphenyl)-3-(2,3-dihydrobenzo[b][1,4]dioxin-6-yl)prop-2-en-1-one* (**26**) Yield 37.89%; m.p.: 155.7–156.1 °C; UPLC purity 95.71%, *t_R_* =9.415 min. ^1^H NMR (400 MHz, DMSO-*d*_6_) δ 12.89 (s, 1H), 8.29 (d, *J* = 8.6 Hz, 1H), 7.85 (d, *J* = 15.4 Hz, 1H), 7.76 (d, *J* = 15.4 Hz, 1H), 7.53 (d, *J* = 2.1 Hz, 1H), 7.39 (dd, *J* = 8.4, 2.1 Hz, 1H), 7.09 (d, *J* = 2.1 Hz, 1H), 7.05 (dd, *J* = 8.6, 2.1 Hz, 1H), 6.94 (d, *J* = 8.4 Hz, 1H), 4.31 (q, *J* = 5.3 Hz, 4H). ^13^C NMR (101 MHz, DMSO-*d*_6_) δ 193.15, 163.01, 146.86, 145.83, 144.13, 140.54, 132.88, 128.33, 124.10, 120.30, 120.04, 119.81, 118.02, 117.97, 117.83, 64.94, 64.41. HRMS: m/z [M + H]^+^ calcd for C_17_H_13_ClO_4_: 317.0575, found: 317.0593.

*(E)-3-(benzo[d][1,3]dioxol-5-yl)-1–(3-bromophenyl)prop-2-en-1-one* (**27**) Yield 22.14%; m.p.: 111.8–112.6 °C; UPLC purity 98.78%, *t_R_* =2.539 min. ^1^H NMR (400 MHz, DMSO-*d*_6_) δ 8.33 (s, 1H), 8.15 (d, *J* = 7.8 Hz, 1H), 7.88 − 7.81 (m, 2H), 7.75 − 7.69 (m, 2H), 7.54 (t, *J* = 7.9 Hz, 1H), 7.36 (dd, *J* = 8.0, 0.9 Hz, 1H), 7.00 (d, *J* = 8.0 Hz, 1H), 6.13 (s, 2H).^13^C NMR (101 MHz, DMSO-*d*_6_) δ 188.01, 150.26, 148.60, 145.37, 140.25, 136.05, 131.42, 131.39, 129.57, 127.87, 126.85, 122.77, 119.93, 108.98, 107.54, 102.18. HRMS: m/z [M + H]^+^ calcd for C_16_H_13_BrO_3_: 330.9968, found: 330.9968.

*(E)-3-(benzo[d][1,3]dioxol-5-yl)-1-(4-chlorophenyl)prop-2-en-1-one* (**28**) Yield 16.27%; m.p.: 125.5–127.1 °C; UPLC purity 99.03%, *t_R_* =2.311 min. ^1^H NMR (400 MHz, DMSO-*d*_6_) δ 8.18 (d, *J* = 8.2 Hz, 2H), 7.82 (d, *J* = 15.5 Hz, 1H), 7.74 − 7.66 (m, 2H), 7.63 (d, *J* = 8.3 Hz, 2H), 7.34 (dd, *J* = 8.0, 1.7 Hz, 1H), 7.00 (d, *J* = 8.0 Hz, 1H), 6.12 (s, 2H). ^13^C NMR (101 MHz, DMSO-*d*_6_) δ 188.26, 150.18, 148.58, 145.00, 138.40, 136.87, 130.81, 129.58, 129.27, 126.64, 120.08, 108.99, 107.46, 102.16. HRMS: m/z [M + H]^+^ calcd for C_16_H_11_ClO_3_: 287.0469, found: 287.0488.

### hMAO inhibition assay

The hMAO inhibition assay was performed as previously described^18^. Adequate amounts of recombinant hMAO-A/B were acquired and adjusted to 12.5 µg/mL or 75 µg/mL for hMAO-A or hMAO-B, respectively, with sodium phosphate buffer (50 mM, pH 7.4). The test compounds were dissolved in DMSO as stock solutions (10 mM) and diluted with sodium phosphate buffer (50 mM, pH 7.4) to the corresponding concentrations. Then the test compounds (20 µL) and hMAO (80 µL) were incubated for 15 min at 37 °C in a flat, black-bottomed 96-well microtest plate in the dark. The reaction was initiated by adding Amplex Red reagent (200 µM, final concentration), horseradish peroxidase (2 U/mL, final concentration), and 2 mM p-tyramine for hMAO-A or 2 mM benzylamine (final concetration) for hMAO-B and incubated at 37 °C for 20 min. Activity was quantified in a multi detection microplate fluorescence reader (SpectraMax M5, Molecular Devices, USA) based on the fluorescence generated (excitation, 545 nm; emission, 590 nm). The specific fluorescence emission was calculated after subtraction of the background activity. The background activity was determined from wells containing all components except the hMAO isoforms, which were replaced by a sodium phosphate buffer solution. The percentage inhibition was calculated by the following expression: (1 – I_Fi_/I_Fc_) × 100, in which I_Fi_ and I_Fc_ are the fluorescence intensities obtained for hMAO in the presence and absence of inhibitors after subtracting the respective background. The synthetic compounds were first screened for the inhibition of hMAO-B at 1 µM, which showed more than 70% enzyme inhibition were then subjected to IC_50_ determination

### Enzyme kinetic study

To evaluate the mode of MAO-B inhibition, the representative compound was evaluated in substrate-dependent kinetic experiments performed as previously described. Sets of Lineweaver–Burk plots were generated. The reciprocal MAO-B activity was plotted against the reciprocal substrate concentration. The initial catalytic rates of human MAO-B were measured at four different concentrations of the substrate benzylamine (1, 2, 4 and 8 mM) in the absence (basal sample) and in the presence of four different concentrations (IC_50_, 3/2 IC_50_, 2 IC_50_ and 5/2 IC_50_) of the inhibitors. The enzymatic reactions and measurements were performed using human MAO-B assay conditions as described above for the determination of IC_50_ values.

### Reversibility study of MAO-B inhibition

To investigate the reversibility of the MAO-B inhibition by the active compounds, we performed time-dependent inhibition experiments using human MAO-B as previously described^11^. R-(–)-deprenyl, rasagiline and safinamide were used as the reference compounds. The hMAO-B enzyme was pre-incubated with the representative compounds at the concentrations of their corresponding IC_80_ for 15 min and the reaction was initiated by adding work solutions as described above. The activity of the enzyme was monitored by fluorescence measurements over a period of 120 min. Control experiments without inhibitors were run simultaneously. The enzymatic activity was determined as described above. The percentage of enzyme activity was plotted against the incubation time to determine time-dependent enzyme inhibition.

### Molecular docking study

A molecular docking study was performed to investigate the possible interaction between the active compounds and hMAOs. The crystal structures of hMAO-B (PDB code 2V61)^19^ and hMAO-A (2Z5X)^20^ were adopted and the compounds were docked into the active sites following the standard dock protocol in the software Molecular Operating Environment (MOE) 2019. For the docking into hMAO-B, nine conserved water molecules were included in the docking process according to previous reports^21^. Default settings were used, unless stated otherwise. The top-ranked poses of the docked compounds were selected and analysed.

### Theoretical calculation of physicochemical parameters

The relevant physicochemical parameters, including molecular weight (M.W), hydrogen bond acceptor and donor (HBA, HBD), number of rotatable bonds (NRB), topological surface area (tPSA) and the octanol/water partition coefficient (LogP), were calculated using the descriptors module in MOE 2019.

### MTT assay

The 3–(4, 5-dimethylthiazol-2-yl)-2,5-diphenyltetrazolium (MTT) assay was performed as previously described^22^. BV2 Cells were plated at 5000 cells per well and allowed to grow at 37 °C overnight. Then the cells were treated with various concentrations of the tested compounds for 24 h at 37 °C in a humidified atmosphere with 5% CO_2_, 0.5% DMSO acted as the vehicle control. The cell culture medium was then removed, followed by the addition of fresh cell culture medium containing 5.0 g/L MTT (20 µL/per well) and incubation at 37 °C in a humidified, 5% CO_2_ atmosphere for 4 h. After this incubation period, the cell culture medium was removed and the formed formazan crystals dissolved in 100% DMSO. The absorbance was measured at 570 nm in a multi-well plate reader. The results are expressed as % relative cell viability compared to DMSO vehicle control.

## Results

### Inhibitory activity of monoamine oxidase

The inhibitory effects of 28 chalcone derivatives on human MAO-B and MAO-A enzymes were screened using a fluorescence-based assay. The tested concentration was 1 µM unless otherwise stated. Irreversible inhibitors R-(–)-deprenyl and rasagiline were used as positive controls for MAO-B inhibition at 0.1 µM. Clorgyline was used as a positive control for MAO-A inhibition. The results are summarised in Table 1.

**Table 1.** MAO inhibitory activities of 1, 4-benzodioxan-substituted chalcone derivatives

Compound	hMAO-A	hMAO-B		Selectivity Index (SI)*^a^*
	IC_50_ (μM)	Inhibition (%)	IC_50_ (μM)	
**1**	>40	41.6% ± 0.003*^b^*	13.04 ± 0.06	>3
**2**	>40	18.1% ± 3.38	N.D.	N.D.
**3**	>40	50.8% ± 9	N.D.	N.D.
**4**	>40	84.9% ± 2.5	0.22 ± 0.005	>186
**5**	>40	75.0% ± 1.7	0.3 ± 0.02	>133
**6**	>40	78.0% ± 0.27	0.34 ± 0.01	>118
**7**	>40	26.0% ± 2.3	N.D.	N.D.
**8**	>40	51.7% ± 6.9	N.D.	N.D.
**9**	>40	86.3% ± 0.9	0.15 ± 0.007	>267
**10**	>40	69.5% ± 0.4	N.D.	N.D.
**11**	>40	22.4% ± 0.9	N.D.	N.D.
**12**	>40	89.2% ± 0.46	0.12 ± 0.01	>333
**13**	>40	92.4% ± 0.59	0.054 ± 0.006	>741
**14**	>40	92.6% ± 0.38	0.081 ± 0.004	>494
**15**	>40	89.2% ± 1	0.13 ± 0.007	>308
**16**	>40	93.3% ± 0.68	0.055 ± 0.002	>727
**17**	>40	93.4% ± 0.57	0.057 ± 0.004	>702
**18**	>40	69.9% ± 7.2	N.D.	N.D.
**19**	>40	68.1% ± 2.0	N.D.	N.D.
**20**	>40	87.5% ± 0.29	0.068 ± 0.007	>588
**21**	>40	76.8% ± 1.1	0.23 ± 0.0005	>174
**22**	>40	95.6% ± 0.21	0.026 ± 0.001	>1538
**23**	>40	88.8% ± 0.75	0.17 ± 0.02	>235
**24**	>40	77.1% ± 3.65	0.25 ± 0.03	>160
**25**	>40	78.4% ± 1.4	0.32 ± 0.05	>125
**26**	>40	62.3% ± 3.2	N.D.	N.D.
**27**	>40	80.4% ± 0.44	0.28 ± 0.002	>143
**28**	>40	57.5% ± 3.2	N.D.	N.D.
R-(-)-deprenyl	N.D.	92% ± 0.738	0.0196*^b^*	N.D.
rasagiline	N.D.	75% ± 0.012	0.069*^c^*	N.D.
clorgyline	86.6%±0.4*^d^*	N.D.	N.D.	N.D.

Data are represented as means ± SEM from at least three independent experiments. *^a^*Selectivity index (SI) = IC_50_ (hMAO-A)/IC_50_ (hMAO-B); *^b^*see Ref. 6; *^c^*see Ref. 7; *^d^*% inhibition at 0.1 μM; N.D., no determined.

Parent chalcone compound **1** exhibited a moderate inhibition effect against hMAO-B (41.6% inhibition at 10 µM), with an IC_50_ of 13.04 µM. The replacement of the B ring of **1** with naphthyl (**2**) or the 1, 3-benzodioxole (**3**) moiety resulted in increased inhibitory activities (18.1% and 50.8% inhibition, respectively), whereas the replacement of the B ring with the 1, 4-benzodioxan moiety, hit potent lead compound **4**, which exhibited a 55-fold higher hMAO-B inhibition (IC_50_ = 0.22 µM) than **1**. These results suggested that the replacement of the B ring of the parent chalcone with fused-ring moieties increases the inhibitory activity and that the 1, 4-benzodioxan substituent is preferable. Thus, we considered compound **4** as a new lead scaffold for further structural modifications.

As for the compounds that possess electron-donating groups -OH and -OCH_3_ at different positions on the A ring, only **9**, which bears a 3′-OH substituent, exhibited higher MAO-B inhibitory activity (IC_50_ = 0.15 µM) than **4**. The introduction of 2′-OH, 4′-OH, 3′-OCH_3_ and 4′-OCH_3_ at the A ring resulted in lower inhibitory activity (**5**, IC_50_ = 0.3 µM; **6**, IC_50_ = 0.34 µM; **7**, 26.0%; **8**, 51.7% inhibition) than **4**. The 2′, 4′-dihydroxy and 3′, 4′-dimethoxy substituted compounds **10** and **11** also showed lower inhibitory activity than **4** (69.5% and 22.4% inhibition, respectively). These results suggested that substitution with the hydroxyl group at the *meta*-position of the A ring is favourable to increase the inhibitory activity of 1, 4-benzodioxan chalcones.

The 1, 4-benzodioxan chalcones that bear electron-withdrawing groups at different positions of the A ring mostly exhibited inhibitory activities against hMAO-B. Introduction of halogen atoms at the *ortho*- and *meta*-position of the A ring resulted in a significantly higher inhibitory potency than **4**. The 2′-F, 2′-Cl, and 2′-Br substituted compounds **12**, **13** and **14** showed a 1.8–4.1-fold higher in inhibitory potency (**12**, IC_50_ = 0.12 µM; **13**, IC_50_ = 0.054 µM; **14**, IC_50_ = 0.081 µM) and the 3′-F, 3′-Br, and 3′-Cl substituted compounds **15**, **16,** and **17** showed 1.7–4-fold higher in inhibitory potency (IC_50_ = 0.13, 0.055, and 0.057 µM, respectively) than **4**. However, the introduction of 4′–Cl and 4′–Br (**18**, and **19**) at the A ring reduced the inhibitory activity compared to **16** and **17** and only 69.9% and 68.1% inhibition were observed at 1 µM, respectably, suggesting that 4′-Cl and 4′-Br were not well tolerated. The introduction of 4′-F maintained potent inhibitory activity (**20**, IC_50_ = 0.068 µM). As the 4′-CF_3_ substituted compound (**21)** only exhibited inhibition potency comparable to that of **4**, we concluded that substitution with halogen atoms at the *ortho*- and *meta*-position of the A ring is favourable to increase the inhibitory activity of 1, 4-benzodioxan chalcones. The introduction of both 4′-F and 3′-Br at the A ring led to the most potent compound (**22)** with an IC_50_ value as low as 0.026 µM, which was comparable to that of safinamide.

The introduction of 3′, 4′-dichloro substituents resulted in less potent inhibitory activity (**23**, IC_50_ = 0.17 µM) than **17**, which might be influenced by the 4′–Cl substitution. The introduction of 2′-OH to compounds **16**–**18** also resulted in lower inhibitory activities (**24**, IC_50_ = 0.25 µM; **25**, IC_50_ = 0.32 µM; **26**, 62.3% inhibition at 1 µM) compared to the corresponding compounds, which indicated that the 2′-OH was not tolerated either. After the replacement of the 1, 4-benzodioxan moiety of **16** and **18** with a 1, 3-dioxaindane moiety, the corresponding compounds **27** and **28** exhibited lower inhibitory activities (**27**, IC_50_ = 0.28 µM; **28**, 57.5% inhibition at 1 µM) than their parent compounds, which further suggested that the 1, 4-benzodioxan moiety is a crucial pharmacophore for this class of inhibitors.

**Figure 2.**
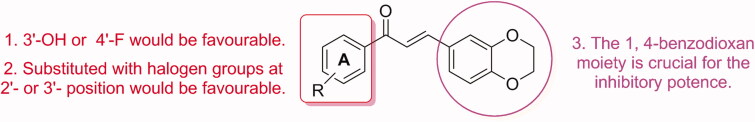
The SAR of 1, 4-benzodioxan-substituted chalcones towards MAO-B inhibition.

An overview of the SAR of 1, 4-benzodioxan-substituted chalcones with respect to MAO-B inhibition is provided in Figure 2.

To hMAO-A, the 1, 4-benzodioxan-substituted chalcone compounds exhibited weak inhibitory activities, with less than 50% inhibition even at 40 µM. A high selectivity index (SI) value was observed regarding the active compounds. The most potent hMAO-B inhibitor (**22)** exhibited an SI value of greater than 1538. Thus, we concluded that the active compounds in the 1, 4-benzodioxan-substituted chalcones are highly selective hMAO-B inhibitors.

### Kinetics study

**Figure 3.**
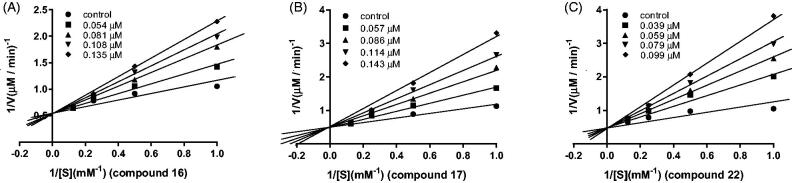
Lineweaver–Burk plots for hMAO-B inhibition by compound **16**, **17**, and **22** (A–C).

Kinetic analyses were carried out with representative compounds **16**, **17** and **22**. The purpose of this experiment was to determine the binding mode of these potent compounds with MAO-B. Lineweaver–Burk graphs were constructed in the absence or presence of inhibitors at various concentrations. The lines are linear and intersect at the y-axis (Figure 3), which suggests that compounds **16**, **17**, and **22** are competitive inhibitors of hMAO-B.

### Reversibility study

To investigate the reversibility of the enzymatic inhibition, we performed time-dependent inhibition studies using representative inhibitors **16**, **17,** and **22** at the concentration of their IC_80_. R-(-)-Deprenyl, rasagiline and safinamide were used as reference inhibitors and evaluated under the same experimental conditions. The hMAO-B activity (% of control) was measured for 120 min in the presence of the tested inhibitors.

**Figure 4.**
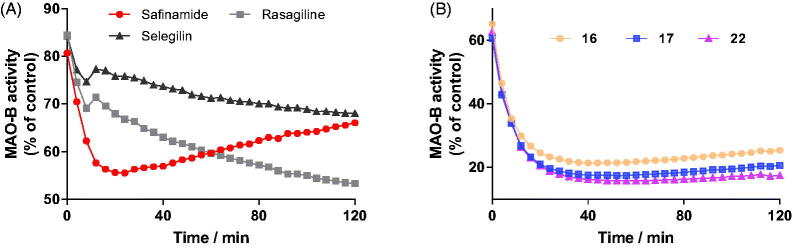
Time-dependent inhibition of hMAO-B by reference compounds R-(–)-deprenyl, rasagiline and safinamide (0.05 μM, 0. 20 μM and 0.06 μM, respectively, A) and test compounds **16**, **17**, and **22** (0.28 μM, 0.28 μM and 0.19 μM, respectively, B). The remaining activity was expressed as % of activity.

When treated with irreversible inhibitors, the residual activity of hMAO-B decayed continuously throughout the incubation period (Figure 4(A)), which indicates that inhibiting hMAO-B by these compounds is irreversible. In the case of the reversible inhibitor safinamide, the residual activity of hMAO-B began to increase after 15 min, indicating that the inhibitor can be replaced by the competing substrate and the enzymatic activity is recovered^11^. Similarly, after incubation with compounds **16**, **17**, and **22** for 20 min, the residual activities of hMAO-B began to increase gradually (Figure 4(B)). These results clearly showed that these active compounds **16**, **17**, and **22** act as reversible hMAO-B inhibitors.

### Molecular docking studies

To investigate the possible interaction mechanism between the active compounds and hMAO-B, we performed a molecular docking study using the docking module in the software MOE. The co-crystal structure of hMAO-B (PDB code 2V61) was employed and the compounds were docked into the active site.

**Figure 5.**
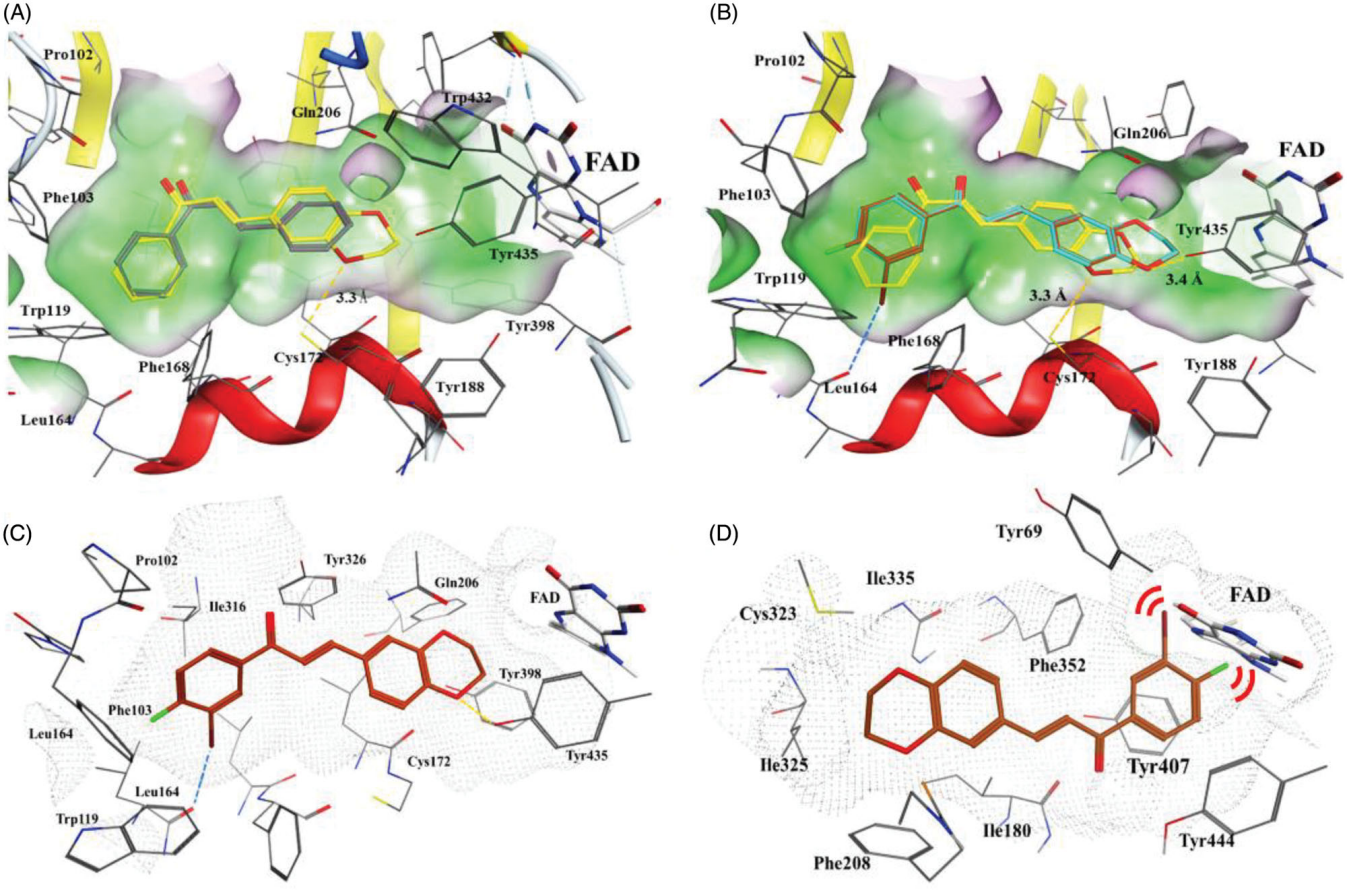
Docking poses of chalcone compounds **1**, **4**, **16**, and **22** in the hMAO-B (PDB code: 2V61) (A–C) or hMAO-A (2Z5X) (D) active site. (A) docking poses of compounds **1 (**gray**)** and **4** (yellow); (B) docking poses of compounds **4** (yellow), **16** (cyan), and **22** (brown); (C) the docking pose of **22** in the active site of hMAO-B; (D) the docking pose of **22** in the active site of hMAO-A. Hydrogen bonds are shown as yellow dotted lines, halogen bonds are shown as blue dotted lines. For clarity, only the relevant residue side chains are shown. FAD is rendered as white sticks. Red bows indicate steric hindrance.

The active site of MAO-B consists of two cavities, the “entrance cavity” and the “substrate cavity”. The entrance cavity is separated from the outer surface by the cavity gating loop (residues 99–110) and the substrate cavity extends from flavin adenine dinucleotide (FAD) to the entrance cavity^23^. Parent chalcone compound **1** adopted an extended pose across both cavities of MAO-B (Figure 5(A)). The A ring occupied the hydrophobic pocket in the entrance cavity and the B ring was directed towards the hydrophobic cavity in front of FAD. The main interaction between them was thought to be hydrophobic. As for compound **4**, the A ring occupied a similar position at the entrance cavity whereas the 1, 4-benzodioxan moiety pointed towards the substrate cavity, the oxygen atom of which established a hydrogen bond with Cys172 (3.3 Å, Figure 5(A)). This may explain the higher inhibitory activity of compound **4** than of compound **1**. Compounds **16** and **22** adopted similar binding poses with hMAO-B, but their oxygen atom at the 1, 4-benzodioxan ring could establish hydrogen bonds with Tyr435 (3.4 Å, Figure 5(B)), instead of Cys172 (> 5.0 Å), which is in line with the binding mode of chromone inhibitors^24^. Additionally, the 3′-Br of compounds **16** and **22** form halogen bonds with the carbonyl oxygen of Leu164 with a bond distance of 3.3 Å, C − Br − O angle of 163°, and Br − O−C angle of 137° with Leu164, which might play a key role in increasing the inhibitory activity of both compounds^25^. Compound **22** exhibited an approximately 2.6-fold greater inhibition activity than **16**, which might be attributed to the additional substitution with 4′-F at the A ring. The 4′-F of **22** was embedded in the local cavity formed by residues Phe103, Phe104, and Trp119 (Figure 5(B–C)), which might strengthen the binding contact with the enzyme and consequently lead to increased inhibitory activity.

We also attempted to explain why the active compounds exhibited weak inhibitory activity against hMAO-A using a docking study. The most active compound (**22)**, which showed the highest SI, was docked into the active site of hMAO-A. hMAO-A has a single substrate cavity of approximately 550 Å^3^, which is shorter in length than that of hMAO-B, which is approximately 700 Å^3^ ^9^. Compound **22** adopted an extended molecular conformation located in the active site of hMAO-A (Figure 5(D)). In contrast to the binding mode with hMAO-B, the 1, 4-benzodioxan ring of **22** located apart from FAD while the A ring and substituents 3′-Br and 4′-F were pressed close to FAD, which indicated a strong steric hindrance effect between them. Therefore, we hypothesise that steric hindrance might be the key factor that prevents the compounds from binding to hMAO-A.

### Evaluation of drug-likeness

As there are specific physicochemical requirements for drugs that act on the central nervous system (CNS), we estimated the drug-likeness of the active compounds using theoretical calculations inMOE. Relevant physicochemical parameters were calculated, including MW, HBA, HBD, NRB, tPSA, Log P and Log BB. The results are summarised in Table 2. According to these data, all selected active compounds were in agreement with Lipinski’s rule of five for drug-likeness without any violation. The calculated values of these parameters were all within the suggested limits set for the development of CNS drugs (MW < 400, HBA ≤ 7, HBD ≤3, NRB < 8, cLogP = 2–5, and tPSA < 70 Å^2^)^26^. All these compounds exhibited a log BB value greater than −1, which indicated that these active compounds might possess good blood-brain barrier (BBB) permeability^27^. We further employed the Cbligand-BBB predictor program^28^ to predict the ability of these compounds to cross the BBB. All the active compounds were predicted to be BBB (+) (Table 2). Similar results were observed for the clinical drugs R-(–)-deprenyl, rasagiline, safinamide and clorgyline.

**Table 2.** Calculation of the drug-like properties of selected active compounds and MAO-B standard inhibitors

entries	M.W	HBA	HBD	NRB	clogP (o/w)	tPSA (Å^2^)	Log BB^a^	lip_violation	Predicted BBB (+/–)^b^
**13**	300.7410	3	0	3	4.2500	35.5300	0.26	0	+
**16**	345.1920	3	0	3	4.4950	35.5300	0.30	0	+
**17**	300.7410	3	0	3	4.2890	35.5300	0.27	0	+
**20**	284.2860	3	0	3	3.8130	35.5300	0.19	0	+
**22**	363.1820	3	0	3	4.6460	35.5300	0.32	0	+
**R-(-)-deprenyl**	187.2860	1	0	5	2.8110	3.2400	0.52	0	+
**rasagiline**	171.2340	1	1	3	2.4620	12.0300	0.34	0	+
**safinamide**	320.3490	3	2	7	2.8540	64.3500	0.38	0	+
**clorgyline**	272.1750	2	0	7	3.7060	12.4700	0.52	0	+
**Suggested limits**	<500	<7	<3	<8	2–5	<90	≥ −1		

*^a^*Log BB = –0.0148 × tPSA + 0.152* clogP + 0.139, see Ref. 25; BBB(±): blood-brain barrier permeability.

### Cell cytotoxicity assay

**Figure 6.**
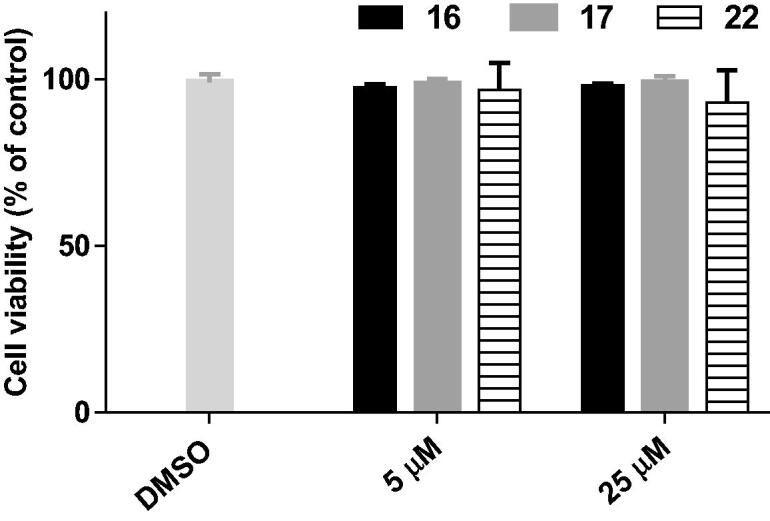
Cell viability of BV2 cells after treatment with compounds **16**, **17**, and **22** at the concentrations of 5 and 25 µM for 24 h.

The cytotoxicity of the potent compounds **16**, **17** and **22** was evaluated in BV2 microglia cells using the MTT assay. For all the tested compounds, no considerable decrease in cellular viability was observed, even at the highest tested concentration (25 µM) (Figure 6). The results suggested that compounds **16**, **17**, and **22** exhibit low cytotoxicity and might have a wide safety window.

## Conclusion

In the current study, a series of 1, 4-benzodioxan-substituted chalcone compounds were designed, synthesised, and evaluated for the inhibition of hMAO-B. Mostshowed potent inhibitory activity and high selectivity. SAR analysis suggested that the 1, 4-benzodioxan moiety is a key pharmacophore and substitution with halogen atoms at the *ortho*- or *meta*-position of the A ring would significantly increase the inhibitory potency of this class of inhibitor. The introduction of 4′-F at the A ring would be favourable. The most potent compound (**22)**, bearing both 3′-Br and 4′-F at the A ring, exhibited an IC50 value of 0.026 µM with an SI value greater than 1538, which was comparable to that of safinamide. Kinetics and reversibility studies confirmed that the representative compounds **16**, **17**, and **22** acted as competitive and reversible inhibitors of hMAO-B. Owing to their promising drug-like properties and low cytotoxicity, we believe that the active 1, 4-benzodioxan-substituted chalcone compounds could serve as valuable drug candidates for further development.
